# Scutellariae Radix and Coptidis Rhizoma Improve Glucose and Lipid Metabolism in T2DM Rats via Regulation of the Metabolic Profiling and MAPK/PI3K/Akt Signaling Pathway

**DOI:** 10.3390/ijms19113634

**Published:** 2018-11-18

**Authors:** Xiang Cui, Da-Wei Qian, Shu Jiang, Er-Xin Shang, Zhen-Hua Zhu, Jin-Ao Duan

**Affiliations:** Jiangsu Collaborative Innovation Center of Chinese Medicinal Resources Industrialization, Nanjing University of Chinese Medicine, 138 Xianlin Road, Nanjing 210023, China; 15951975518@163.com (X.C.); qiandw@njucm.edu.cn (D.-W.Q.); shex@sina.com (E.-X.S.); 18913133908@163.com (Z.-H.Z.)

**Keywords:** SR, CR, Compatibility, T2DM, metabolic profiling, MAPK/PI3K/Akt signaling pathway

## Abstract

*Aim* Scutellariae Radix (SR) and Coptidis Rhizoma (CR) have often been combined to cure type 2 diabetes mellitus (T2DM) in the clinical practice for over thousands of years, but their compatibility mechanism is not clear. Mitogen-activated protein kinase (MAPK) signaling pathway has been suggested to play a critical role during the process of inflammation, insulin resistance, and T2DM. This study was designed to investigate their compatibility effects on T2DM rats and explore the underlying mechanisms by analyzing the metabolic profiling and MAPK/PI3K/Akt signaling pathway. *Methods* The compatibility effects of SR and CR were evaluated with T2DM rats induced by a high-fat diet (HFD) along with a low dose of streptozocin (STZ). Ultra performance liquid chromatography-quadrupole time-of-flight mass spectrometry (UPLC-Q-TOF/MS) was performed to discover potential biomarkers. The levels of pro-inflammatory cytokines; biochemical indexes in serum, and the activities of key enzymes related to glycometabolism in liver were assessed by ELISA kits. qPCR was applied to examine mRNA levels of key targets in MAPK and insulin signaling pathways. Protein expressions of p65; p-p65; phosphatidylinositol-4,5-bisphosphate 3-kinase (PI3K); phosphorylated-PI3K (p-PI3K); protein kinase B (Akt); phosphorylated Akt (p-Akt) and glucose transporter 2 (Glut2) in liver were investigated by Western blot analysis. *Results* Remarkably, hyperglycaemia, dyslipidemia, inflammation, and insulin resistance in T2DM were ameliorated after oral administration of SR and CR, particularly their combined extracts. The effects of SR, CR, low dose of combined extracts (LSC) and high dose of combined extracts (HSC) on pro-inflammatory cytokine transcription in T2DM rats showed that the MAPK pathway might account for the phenomenon with down-regulation of MAPK (P38 mitogen-activated protein kinases (P38), extracellular regulated protein kinases (ERK), and c-Jun N-terminal kinase (JNK)) mRNA, and protein reduction in p-P65. While mRNA levels of key targets such as insulin receptor substrate 1 (IRS1), PI3K, Akt2, and Glut2 in the insulin signaling pathway were notably up-modulated, phosphorylations of PI3K, Akt, and expression of Glut2 were markedly enhanced. Moreover, the increased activities of phosphoenolpyruvate carboxykinase (PEPCK), fructose-1,6-bisphosphatase (FBPase), glucose 6-phosphatase (G6Pase), and glycogen phosphorylase (GP) were highly reduced and the decreased activities of glucokinase (GK), phosphofructokinase (PFK), pyruvate kinase (PK), and glycogen synthase (GS) in liver were notably increased after treatment. Further investigation indicated that the metabolic profiles of plasma and urine were clearly improved in T2DM rats. Fourteen potential biomarkers (nine in plasma and five in urine) were identified. After intervention, these biomarkers returned to normal level to some extent. *Conclusion* The results showed that SR, CR, and combined extract groups were normalized. The effects of combined extracts were more remarkable than single herb treatment. Additionally, this study also showed that the metabonomics method is a promising tool to unravel how traditional Chinese medicines work.

## 1. Introduction

Type 2 diabetes mellitus (T2DM), characterized by increased blood glucose level resulting from disturbances of insulin secretion, insulin action or both, is a complex disorder influenced by both lifestyle and genetic factors. T2DM and its serious complications such as nerve damage [1], kidney disease [2], and cardiovascular disease [3,4] have significantly increased society’s medical burden over the past few decades and it is estimated that their global numbers in adults will rise to 592 million by 2035 [5]. Thus, its prevalence has become a major public health issue throughout the world, especially in developing countries.

Currently, drugs clinically used to treat T2DM include insulin sensitizers, insulin secreting drugs, insulin, etc. Although current diabetes treatments have exhibited some success in lowering blood glucose levels, their effects are not always sustained and their use may be associated with undesirable side effects such as hypoglycemia [6], gastrointestinal discomfort [7], etc. So it is urgent to discover new and effective drugs with fewer side effects to cure T2DM. Luckily, successful therapies for T2DM have been available from TCM practitioners. Thus, many researchers have focused on developing potent therapies and drugs from Chinese herbs with fewer side effects on T2DM patients.

SR, the dry root of *Scutellaria baicalensis* Georgi, with a number of biological activities such as anti-inflammation [8], anti-cancer [9], and anti-oxidation [10], has been used to treat various types of diseases. The major pharmacologically-active components of SR are flavonoids such as baicalin, wogonoside, baicalein, and wogonin. Accumulating researches have revealed that baicalin can significantly improve insulin resistance [11,12] and suppress gluconeogenesis [13]. CR, the dried rhizome of *Coptis chinensis* Franch, mainly containing alkaloids such as berberine, coptisine, and palmatine, has been used in China for thousands of years to treat diarrhea. Moreover, recent researches have showed that CR has anti-bacterial [14], anti-cancer [15] activities, etc. Especially, berberine has been shown to possess remarkable effects on lowering blood glucose and promoting the secretion of insulin [16,17]. In China, SR and CR are often used together at a ratio of 1:1 to obtain a synergistic effect for treating diabetes and its complications, yet the underlying mechanism on the therapy of T2DM remains unclear.

Due to insulin resistance, the liver excessively releases glucose into the blood as a result of increased glycogenolysis and gluconeogenesis. Besides, decreased glycolysis and glycogenesis lead to reduced consumption of blood glucose, contributing to hyperglycemia eventually [18]. Inflammatory cytokines such as TNF-α and IL-6 promote the development of insulin resistance, and a recent study has reported that anti-inflammation agents showed efficacy in reducing blood glucose level [19]. Moreover, T2DM is often present for years before becoming clinically apparent. Current clinical predictors such as fasting blood glucose are helpful in gauging diabetes risk, but they only reflect extant disease and provide little additional insight regarding pathophysiologic mechanisms. Earlier identification of individuals at risk is necessary and emerging technologies have made it possible through metabolomics. Among the varieties of analytical platforms used for metabonomic analysis, the use of ultra performance liquid chromatography-quadrupole time-of-flight mass spectrometry (UPLC-Q-TOF/MS) is steadily increasing due to its better reproducibility and detection limits, and increased chromatographic resolution, which can assess all metabolites in biological samples and provide insights into the holistic efficacy of TCMs. Besides, the extent of metabolic changes and types of metabolites could be applied as good markers during insulin resistance [20].

Thus, in this study, T2DM rats induced by HFD along with a low dose of STZ were used to assess the efficacy of individual and combined extracts. The levels of biochemical indexes, pro-inflammatory cytokine, key targets in MAPK, and insulin signaling pathways as well as the activities of key enzymes were determined. To elucidate the mechanism, the metabolic changes in plasma and urine from T2DM rats based on UPLC-Q-TOF/MS were investigated. Potential biomarkers and metabolic pathways were also identified. These data would provide sound scientific evidence for the clinical treatment of T2DM.

## 2. Results

### 2.1. Ameliorative Effect of SR, CR, and Combined Extracts on Hyperglycemia, Dyslipidemia, and Insulin Resistance in T2DM Rats

**Figure 1:**
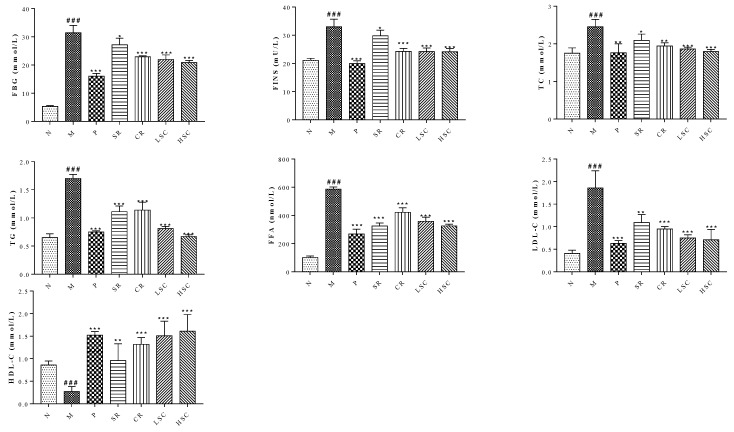
The levels of fasting blood glucose (FBG), fasting insulin (FINS), total cholesterol (TC), triglyceride (TG), free fatty acid (FFA), high-density lipoprotein cholesterol (HDL-C), low-density lipoprotein cholesterol (LDL-C) in serum of the normal group (N), model group (M), and groups gavaged with metformin (P), scutellaria Radix (SR), coptidis Rhizome (CR), low dose of combined extracts group (LSC) and high dose of combined extracts (HSC). The values were shown as mean ± SD. # *p* < 0.05, ## *p* < 0.01, ### *p* < 0.001 vs. normal group; * *p* < 0.05, ** *p* < 0.01, *** *p* < 0.001 vs. model group. Data were analyzed by One-way-ANOVA.

To evaluate the compatibility effects of SR and CR, a favorable T2DM rat model with a notable glycolipid metabolism disorder was established by HFD along with low dose of STZ. The significant increase of FBG, FINS, TG, TC, LDL-C, and FFA levels in T2DM rats was observed. By contrast, compared with T2DM rats, the levels of the above biochemical factors were decreased to a range from 51.1% to 85.4% after oral administration of individual and combined extracts. Furthermore, LSC or HSC extracts exerted better effects than the single drug, and the levels of these biochemical indexes declined remarkably after treatment. Metformin demonstrated a similar regulation as HSC in T2DM rats (Figure 1). These results indicated the better effects of combined extracts on the regulation of glucose and lipid homeostasis.

### 2.2. Pathological Assessment of Some Tissues Related to Insulin Resistance

**Figure 2:**
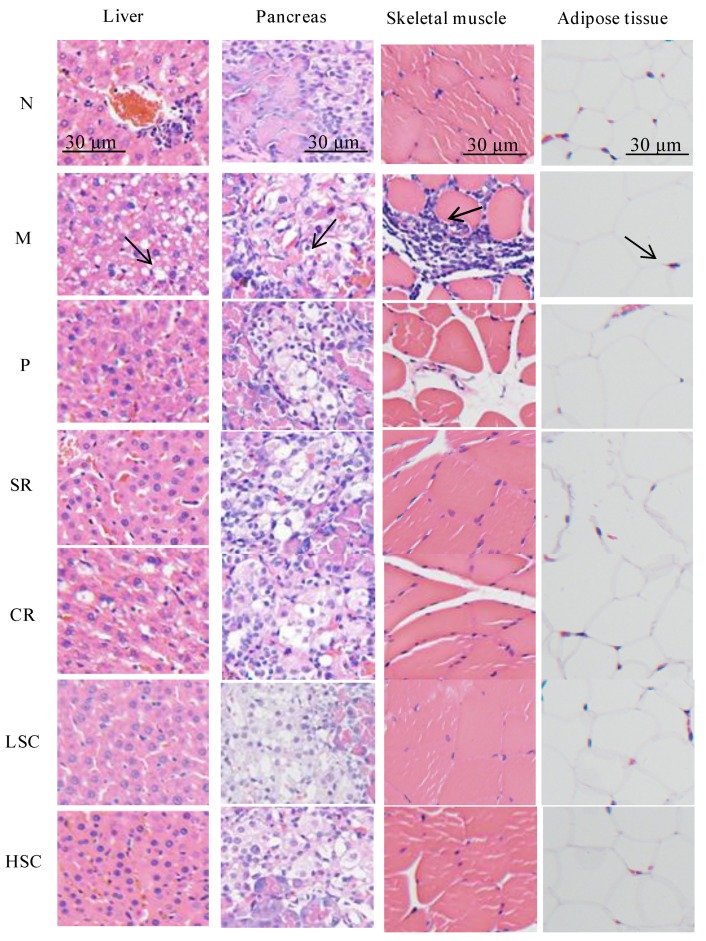
Histopathological observation of liver, pancreas, skeletal muscle and adipose tissue in the normal group (N), model group (M), and groups gavaged with metformin (P), scutellaria Radix (SR), coptidis Rhizome (CR), low dose of combined extracts group (LSC) and high dose of combined extracts (HSC). Liver tissues of M: notable changes with severe fatty degeneration can been seen as shown by the black arrow; Pancreas of M: severe atrophy of pancreatic islet cells is shown by the black arrow; Skeletal muscle of M: notably inflammatory cells infiltration is indicated by the black arrow; Adipose tissues of M: mean adipocyte diameter was seriously increased, as indicated by the black arrow. Samples were stained with H&E and photographed at 400× magnification.

The liver tissues of normal rats exhibited normal cellular structure with neat liver lobule, liver cords, liver sinusoid, and a clear three pipeline structure of the portal area. While notable changes with severe fatty degeneration were observed in the liver tissues of T2DM rats, this indicated that the model had been successfully established. Compared to model rats, SR and CR group rats showed moderate fatty degeneration, while LSC, HSC, and P group rats showed mild fatty degeneration by HE staining observation. The islet cells of T2DM rats showed severe atrophy. After treatment, SR and CR group rats showed moderate atrophy, however, LSC, HSC, and P group rats showed mild atrophy. The mean adipocyte diameter of T2DM rats compared with normal rats was seriously increased. After treatment, the mean adipocyte diameter was decreased and HSC showed a normalized effect. The skeletal muscle of T2DM rats was notably infiltrated with inflammatory cells, but P, SR, CR, LSC, and HSC group rats were normalized (Figure 2). The histological examination results were consistent with biochemical data, which further confirmed the synergistic effect of SR and CR.

### 2.3. SR, CR, and Combined Extracts Alleviated Inflammation by Regulation of Pro-Inflammatory Cytokine Expressions through MAPK Signaling Pathway

**Figure 3:**
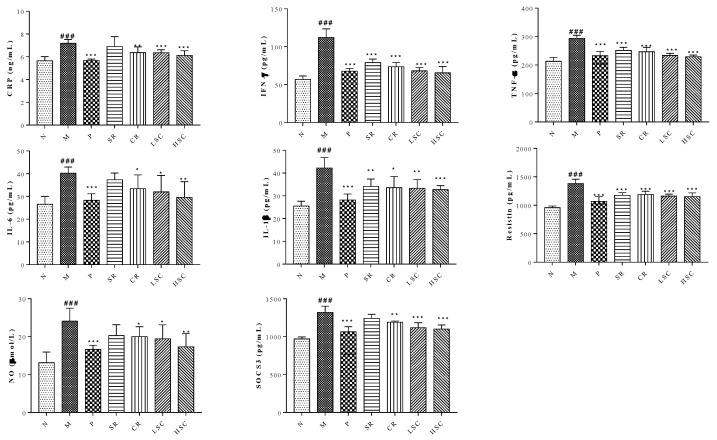
Serum contents of C-reaction protein (CRP), interferon gamma (IFN-γ), tumor necrosis factor-α (TNF-α), interleukin-6 (IL-6), interleukin-1β (IL-1β), resistin, nitric oxide (NO) and suppressor of cytokine signaling 3 (SOCS3) in the normal group (N), model group (M), and groups gavaged with metformin (P), scutellaria Radix (SR), coptidis Rhizome (CR), low dose of combined extracts group (LSC) and high dose of combined extracts (HSC). The values were shown as mean ± SD. # *p* < 0.05, ## *p* < 0.01, ### *p* < 0.001 vs. normal group; * *p* < 0.05, ** *p* < 0.01, *** *p* < 0.001 vs. model group. Data were analyzed by One-way-ANOVA.

**Figure 4:**
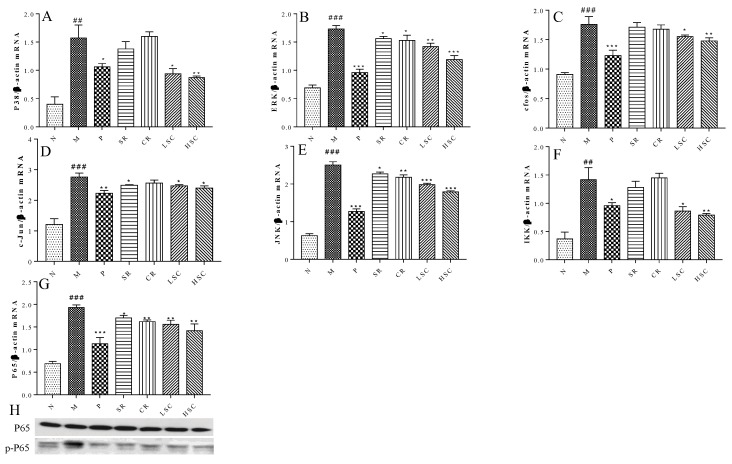
The effect of metformin (P), scutellaria Radix (SR), coptidis Rhizome (CR), low dose of combined extracts group (LSC) and high dose of combined extracts (HSC) on MAPK signaling pathway. (**A**–**G**) mRNA expression levels of c-jun N-terminal kinase (JNK), p38 mitogen-activated protein kinases (P38), extracellular regulated protein kinases (ERK), c-fos, c-jun, Inhibitor of nuclear factor kappa-B kinase (IKK) and P65 in liver of the N, M, P, SR, CR, LSC, and HSC by qPCR; (**H**) Protein levels of P65, phosphorylated P65 (p-P65) in liver of the N, M, P, SR, CR, LSC, and HSC by Western blot. The values are shown as mean ± SD. # *p* < 0.05, ## *p* < 0.01, ### *p* < 0.001 vs. normal group; * *p* < 0.05, ** *p* < 0.01, *** *p* < 0.001 vs. model group. Data were analyzed by One-way-ANOVA.

Recently, many studies have shown that pro-inflammatory macrophage accumulation in liver could trigger chronic low-grade inflammation that promoted the development of insulin resistance [21]. On day 30 after STZ injection, levels of CRP, IFN-γ, TNF-α, IL-6, resistin, SOCS3, IL-1β, and NO in serum were significantly increased in the model group compared to those in the normal group. However, the levels of these pro-inflammatory cytokines were reduced to a range from 96.0% to 58.4% after oral administration of metformin, SR, CR, LSC or HSC (Figure 3). MAPK pathway plays an important role in the inflammatory process [22], so the key targets in this pathway were investigated in this study. As the qPCR results show in Figure 4A–G, mRNA expressions of P38, ERK, c-jun, c-fos, JNK, IKK and P65 were significantly increased in the model group. With the treatment of metformin, SR, CR, LSC, and HSC, mRNA expressions of the above major targets in MAPK pathway were remarkably decreased to a range from 87.9% to 68.8%. Besides, the effects of LSC and HSC were more significant than SR and CR, while metformin showed a similar effect to HSC. NF-κB/p65 is a key transcriptional factor of inflammation regulated gene expression of pro-inflammatory cytokines. Further studies indicated that P65 was activated by phosphorylation in the model group, which could be suppressed by metformin, SR, CR, LSC, and HSC (Figure 4H).

### 2.4. SR, CR and Combined Extracts Activated Insulin Signaling Pathway in Liver

**Figure 5:**
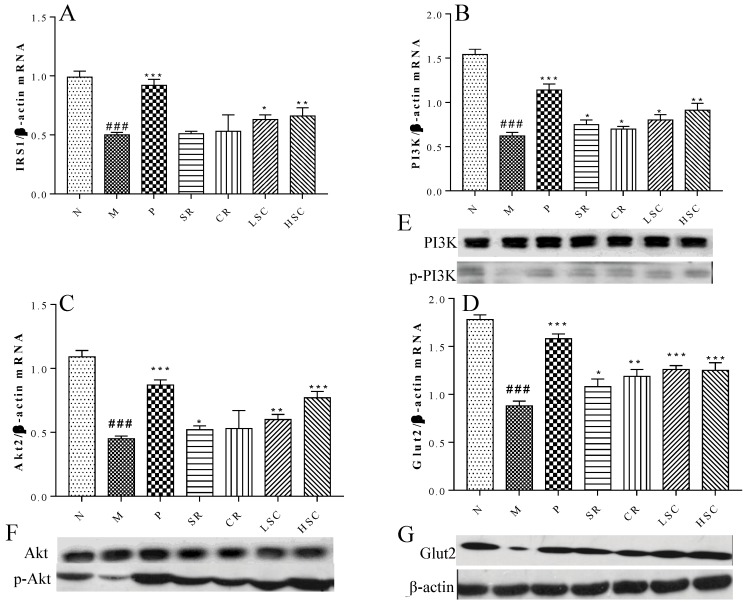
The effect of metformin (P), scutellaria Radix (SR), coptidis Rhizome (CR), low dose of combined extracts group (LSC) and high dose of combined extracts (HSC) on insulin signaling pathway. (**A**–**D**) mRNA expression levels of Insulin receptor substrate 1 (IRS1), Phosphatidylinositol-4,5-bisphosphate 3-kinase (PI3K), Protein kinase B (Akt2) and Glucose transporter 2 (Glut2) in liver of the N, M, P, SR, CR, LSC, and HSC by qPCR; (**E**–**G**) Protein expression levels of PI3K, phosphorylated PI3K (p-PI3K), Akt2, phosphorylated Akt2 (p-Akt2), Glut2 in liver of the N, M, P, SR, CR, LSC, and HSC by Western blot. The values are shown as mean ± SD. # *p* < 0.05, ## *p* < 0.01, ### *p* < 0.001 vs. normal group; * *p* < 0.05, ** *p* < 0.01, *** *p* < 0.001 vs. model group. Data were analyzed by One-way-ANOVA.

Insulin resistance was derived from systemic inflammation in T2DM [23]. Our researches suggested that LSC and HSC could markedly inhibit inflammation in liver, and subsequent investigation was carried out to determine whether this action contributed to improve insulin signaling. As the qPCR results show in Figure 5A–D, mRNA expressions of IRS1, PI3K, Akt2, and Glut2 were markedly decreased in T2DM rats. Additionally, protein levels of p-PI3K, p-Akt, and Glut2 were notably lower in T2DM rats than those in normal rats (Figure 5E–G). After treatment, mRNA expressions of IRS1, PI3K, Akt2, and Glut2 were remarkably increased to a range from 102% to 171% and protein levels of p-PI3K, p-Akt and Glut2 were notably increased. However, protein levels of PI3K, Akt were not significantly different among groups. Furthermore, LSC and HSC were more effective than individual SR and CR.

### 2.5. SR, CR and Combined Extracts Suppressed Hepatic Glucose Output by Inhibiting Gluconeogenesis and Glycogenolysis as Well as Promoting Glycolysis and Glycogenesis

**Figure 6:**
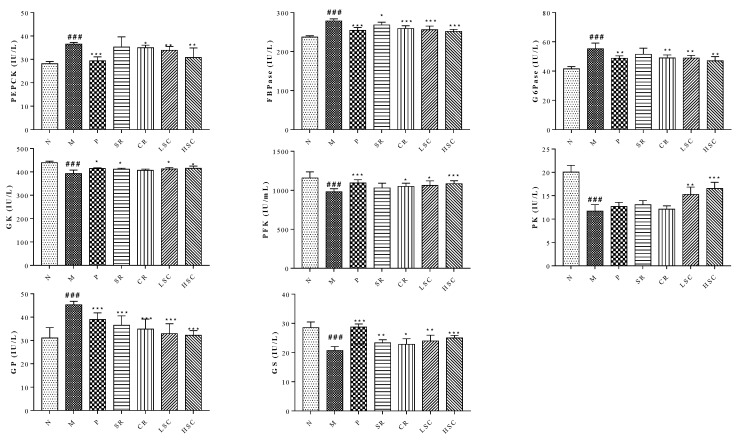
Activities of glucokinase (GK), phosphofructokinase (PFK), pyruvate kinase (PK), phosphoenolpyruvate carboxykinase (PEPCK), fructose-1,6-bisphosphatase (FBPase), glucose 6-phosphatase (G6Pase), glycogen synthase (GS) and glycogen synthase (GP) in livers of the normal group (N), model group (M), and groups gavaged with metformin (P), scutellaria Radix (SR), coptidis Rhizome (CR), low dose of combined extracts group (LSC) and high dose of combined extracts (HSC). The values were shown as mean ± SD. # *p* < 0.05, ## *p* < 0.01, ### *p* < 0.001 vs. normal group; * *p* < 0.05, ** *p* < 0.01, *** *p* < 0.001 vs. model group. Data were analyzed by One-way-ANOVA.

Gluconeogenesis and glycogenolysis are two major pathways for endogenous glucose production [24,25]. PEPCK, FBPase, G6Pase, and GP are the rate-limiting enzymes controlling gluconeogenesis and glycogenolysis in the liver, respectively. The activities of these enzymes were increased in T2DM rats compared to normal rats but the increased activities were remarkably decreased to a range from 96.4% to 71.1% by SR and CR, especially by their combined extracts (Figure 6).

Glycolysis is regarded as a feeder pathway that prepares glucose for further catabolism and energy production. Besides, glucose storage in the form of glycogen could suppress hepatic glucose output to maintain blood glucose homeostasis. GK, PFK, PK, and GS are the rate-limiting enzymes controlling glycolysis and glycogen synthesis, respectively [26]. However, the activities of GK, PFK, PK, and GS were decreased in T2DM rats. After treatment, the decreased activities were increased to a range from 111.7% to 141.5% by SR and CR, especially their combined extracts (Figure 6).

### 2.6. Intervention Effects of SR, CR and Combined Extracts on the Metabolic Profiling of T2DM Rats

**Figure 7:**
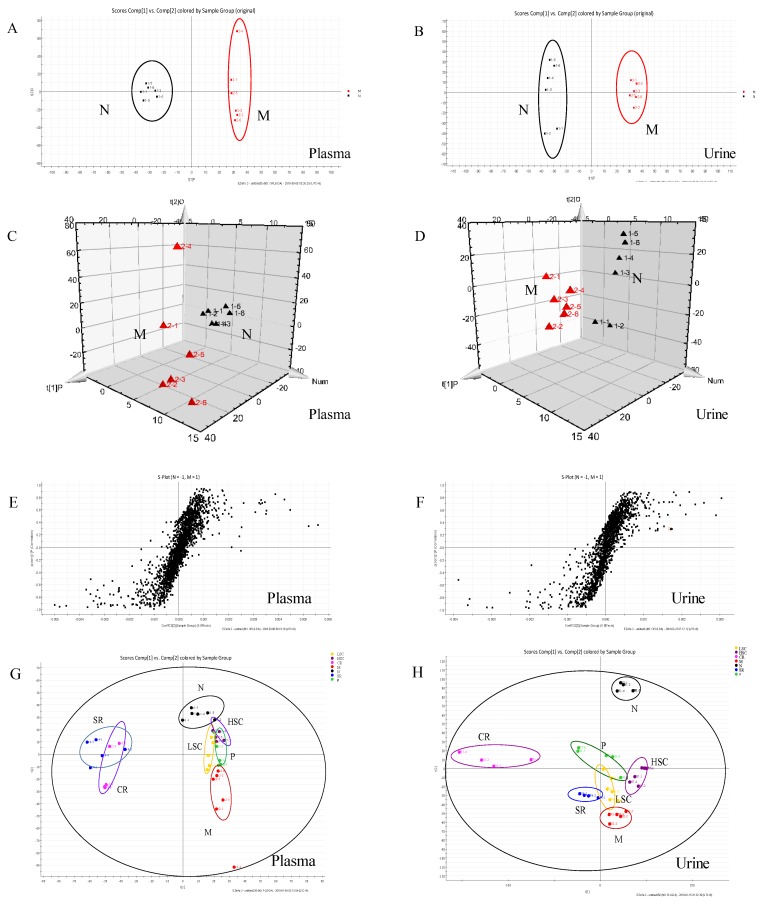
PCA model results between normal (N) and T2DM (M) rats in negative mode ((**A**) 2-D plot of plasma; (**B**) 2-D plot of urine). 3D PLS-DA scores plot of LC–MS spectral data ((**C**) plasma; (**D**) urine). S-plot of OPLS-DA model for M vs. N group ((**E**) plasma; (**F**) urine). PCA analytical results from N and M rats treated with metformin (P), scutellaria Radix (SR), coptidis Rhizome (CR), low dose of combined extracts group (LSC) and high dose of combined extracts (HSC) at negative mode ((**G**) plasma; (**H**) urine).

**Figure 8:**
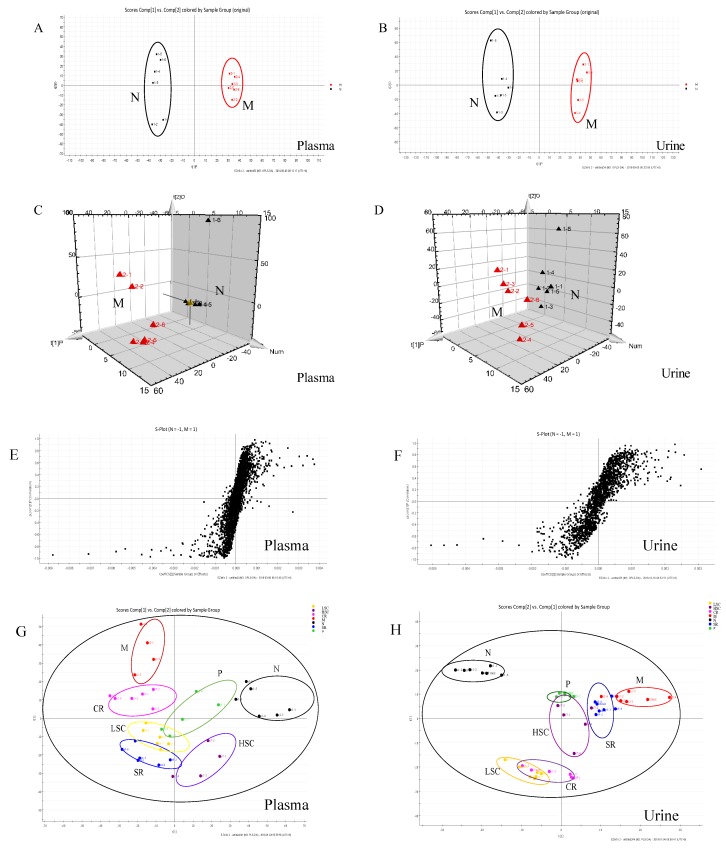
PCA model results between normal (N) and T2DM (M) rats in positive mode ((**A**) 2-D plot of plasma; (**B**) 2-D plot of urine). 3D PLS-DA scores plot of LC-MS spectral data ((**C**) plasma; (**D**) urine). S-plot of OPLS-DA model for M vs. N group ((**E**) plasma; (**F**) urine). PCA analytical results from N and M rats treated with metformin (P), scutellaria Radix (SR), coptidis Rhizome (CR), low dose of combined extracts group (LSC) and high dose of combined extracts (HSC) at positive mode ((**G**) plasma; (**H**) urine).

In this study, UPLC-Q-TOF-MS/MS was applied to generate metabolic profiles of plasma and urine from normal and T2DM rats before and after oral administration of SR, CR, LSC, and HSC in the negative and positive ESI modes. The metabolomics profiles of N and M groups were separated by PCA analysis and orthogonal partial least squares discriminant analysis (OPLS-DA). To investigate the amelioration of SR, CR, LSC, and HSC for curing T2DM, another PCA model was built. The variations of plasma and urine metabolic profiling of SR, CR, LSC, and HSC were restored to the levels of the normal group (Figure 7 and Figure 8). Furthermore, the relative quantities of nine potential biomarkers in plasma and five in urine were significantly regulated by LSC and HSC. There were no obvious effects on the levels of xanthosine and N-acetyl-L-tyrosine for the SR group. The levels of xanthosine and hippuric acid were not regulated by the CR group. It was interesting to note that the levels of xanthosine were neither affected by SR nor CR, but by their combination. The contents of the potential biomarkers in Table S2 were considered as biomarkers for the effects of treatment.

**Figure 9:**
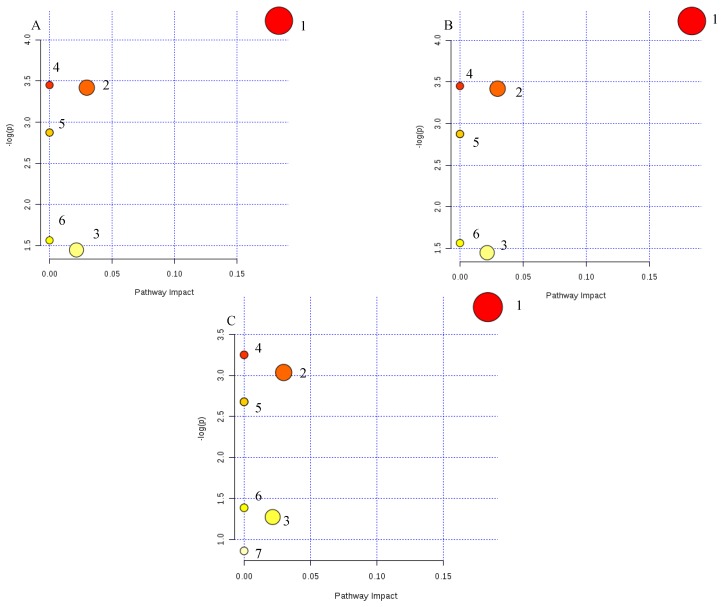
Metabolic pathways involved in potential markers in plasma and urine. (**A**) Scutellaria Radix group (SR); (**B**) Coptidis Rhizome group (CR); (**C**) metformin group (P), Low dose of combined extracts group (LSC) and High dose of combined extracts group (HSC). (**1**) Glycerophospholipid metabolism, (**2**) primary bile acid biosynthesis, (**3**) pyrimidine metabolism, (**4**) linolenic acid metabolism, (**5**) alpha-linolenic acid metabolism, (**6**) arachidonic acid metabolism, (**7**) purine metabolism.

**Figure 10:**
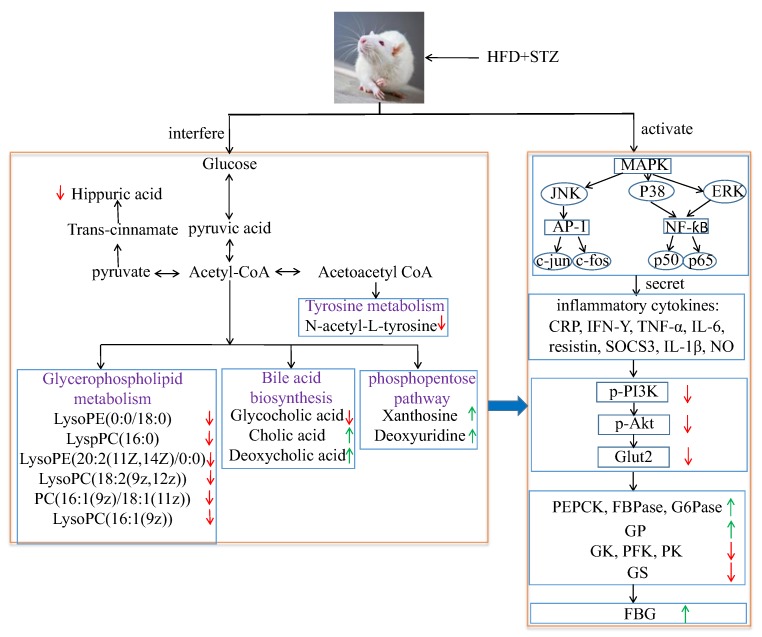
Correlation networks of main potential biomarkers in response to T2DM rats. Compared with the normal group, the red arrows represent the decrease of the contents of endogenous metabolites, while the green arrows represent the increase of the contents of endogenous metabolites.

The metabolic pathway was established by importing the potential metabolites into the web-based database MetPA. The pathway impact value calculated from pathway to topology analysis with MetPA above 0.1 was screened out as the potential target pathway. Here in Figure 9, glycerophospholipid metabolism with the impact values of 0.18 were filtered out as the most important metabolic pathways. Moreover, a correlation network of potential metabolites related to T2DM was exhibited in Figure 10. Our results also indicated that administration of LSC and HSC could more effectively modify these biomarkers and their related metabolic pathways including purine metabolism in which SR and CR could not be involved in T2DM rats.

## 3. Discussion

SR and CR, especially their combined extract, have been clinically used to treat T2DM for thousands of years; however, their compatibility mechanism remains unknown. Thus, in this study, the amelioration of SR, CR and their combined extracts on T2DM was first investigated, and their compatibility mechanism was further unraveled by the metabolomics and MAPK/PI3K/Akt signaling pathway. Importantly, the induction of the T2DM animal model is widely desired. The genetically modified mice and STZ-induced T2DM rat models have ever been established, however, neither these models nor stable animal models are appropriate because of ignoring dietary factors. Therefore, a suitable and stable T2DM rat model using HFD along with low dose of STZ was successfully established in our study. The levels of FBG, TG, TC, LDL-C, and FFA were remarkably increased, while the level of HDL-C was notably decreased in T2DM rats, which was similar to human T2DM sharing common symptoms (Figure 1).

Additionally, accumulating studies indicated that chronic inflammation was a major contributor to insulin resistance and T2DM [27,28,29,30]. Pro-inflammatory cytokines, particularly TNF-α, could enhance the serine phosphorylation of insulin receptor substrate-1, which induced insulin resistance. Some studies have shown glucose lowering effects of specific TNF-α or IL-1β inhibitors [31,32]. As expected, SR and CR could markedly ameliorate inflammation, insulin resistance, hyperglycemia, and hyperlipemia of T2DM rats. Furthermore, their compatibility (LSC and HSC) was more effective than single drug (Figure 1 and Figure 3).

MAPK signaling pathway played a crucial role in inflammation [33]. Especially, NF-κB was a principle factor in regulating inflammatory mediators during the inflammatory process. The stimulation of NF-κB increased the levels of downstream inflammatory factors such as TNF-α, IL-1β, and IL-6, which promoted the insulin resistance in the liver. mRNA expressions of key targets in MAPK pathway were significantly decreased after treatment. Additionally, protein expression of p-P65 was also decreased. Current data elucidated that LSC and HSC could exert better anti-inflammatory effects via blocking of the MAPK pathway (Figure 4).

The amelioration of inflammation might contribute to activating insulin signaling in liver. Remarkably, LSC and HSC inhibited inflammation in liver, but it was tempting to know whether this action was responsible for improving the insulin signaling pathway. The levels of Glut2, p-PI-3K, and p-Akt are major indexes that reflect the activity of the insulin signaling pathway, play a vital role in insulin-activated glucose uptake in liver, and inhibit glucose release from hepatocytes. As expected, LSC and HSC markedly up-regulated the mRNA expressions of IRS1, PI3K, Akt2, and Glut2, while protein expressions of p-PI3K, p-Akt, and Glut2 were also enhanced. These data suggested that LSC and HSC could overcome the impairment in PI3K/Akt insulin signaling pathway, contributing to the improvement of glucose disposal in T2DM rats (Figure 5).

Subsequently, the influence of activated insulin signaling pathway on glycogenolysis, glycogen synthesis, gluconeogenesis, and glucose metabolism in liver was investigated. The liver is crucial for the maintenance of normal glucose homeostasis—it produces glucose during fasting and stores glucose postprandially. Insulin could inhibit glycogenolysis, stimulate glycogen synthesis, reduce gluconeogenesis, and increase glucose metabolism. However, these hepatic processes were dysregulated in T2DM because the liver became insulin resistant. Gluconeogenesis and glycogenolysis constituted two major pathways for endogenous glucose production. Gluconeogenesis involved the production of glucose from non-carbohydrate precursors such as glycerol, glycogenic amino acid, and lactate during non-availability of dietary glucose, or glycogen stored in the liver was degraded to glucose by glycogenolysis. PEPCK, FBPase, and G6Pase acted irreversibly in the gluconeogenesis pathway. FBPase inhibitors and a decrease in transcription of PEPCK and G6Pase genes could reduce excessive endogenous glucose production in T2DM [34,35]. GP regulated hepatic glycogenolysis and GP inhibitor could lower blood glucose [36]. GS modulated hepatic glycogen synthesis and GSK-3 specific inhibitor could enhance glycogen synthesis by increasing GS activity [37]. GK mainly expressed in hepatocytes had effects on regulating blood glucose level in vivo [38]. Previous study also found that the ability of glucose metabolism in T2DM was reduced when the syntheses of PFK and PK were decreased [39]. In this work, owing to the amelioration of the insulin signaling pathway, PEPCK, FBPase, G6Pase, and GP activities were notably decreased, while prominent increased activities of GK, PFK, PK, and GS were detected after treatment. The above data indicated that the activities of key enzymes related to glycometabolism were modulated to notably lower FBG by SR and CR, especially by their compatibility (Figure 6).

As T2DM was a metabolic disease, the effects of SR, CR, LSC, and HSC on metabolic disorders in T2DM rats were further investigated to depict more insight into their compatibility mechanism. Metabolite profiles are closely related to the development of T2DM [40,41,42,43]. Among the most recent investigations on metabolic dysfunctions and plasma metabolites in T2DM, plasma level of bile acids have been confirmed to be closely associated with T2DM [44,45]. Moreover, metabolites of plasma and urine could reflect the changes of systemic physiology. Therefore, based on the comparative analysis of plasma and urine metabolomics in normal and T2DM rats, potential biomarkers and the correlated pathways could be discovered, which would be helpful to clarify the compatibility mechanism of SR and CR in improving metabolic disturbance of T2DM rats. In this study, plasma metabolomics of T2DM rats suggested that cholic acid and deoxycholic acid (secondary bile salts) levels were obviously increased, while the glycocholic acid (conjugated primary bile salts) level was dramatically decreased. Bile acids are major components of bile formed from cholesterol through various enzymatic reactions in hepatocytes. Primary bile acids are synthesized mainly by conjugation with taurine or glycine through the terminal side-chain carboxylic group presenting in the bile acid structure. Bile salt hydrolases, which are produced by many members of gut microbiota, could remove the conjugated amino acid from the primary bile salt to cholate and deoxycholate. Bile acids could exert effects on glucose metabolism via activation of bile acid receptors and G-protein-coupled membrane bound receptors (TGR5) [46,47,48,49,50,51]. TGR5 was expressed in cells of the hematopoietic system such as monocytes and macrophages and conferred anti-inflammatory properties in vitro and in vivo, decreasing cytokine production in monocytes, macrophages and kupffer cells [52,53]. Recent works revealed that the immune-suppressive actions of TGR5 in macrophages contributed to the prevention of the T2DM process [48]. Thus, we speculated that glycocholic acid might be an endogenous TGR5 ligand, while cholic acid and deoxycholic acid were ligand inhibitors. So glycocholic acid could suppress inflammation by TGR5 activation. Both P38 and ERK were significantly stimulated by deoxycholic acid in a prior study [54]. Supplied with deoxycholic acid, serum glucose and triglyceride levels were increased in mice [55]. After treatment, the levels of cholic acid and deoxycholic acid were markedly decreased and glycocholic acid was notably increased. Furthermore, the combination of SR and CR was more effective than the single drug (Table S2).

Additionally, the glycerophospholipid metabolism was the most important metabolic pathway. The decreased levels of glycerophospholipid metabolites including LysoPE(0:0/18:0), LyspPC(16:0), LysoPE(20:2(11Z,14Z)/0:0), LysoPC(18:2(9z,12z)), PC(16:1(9z)/18:1(11z)), and LysoPC(16:1(9z)) indicated a marked perturbation in the glycerophospholipid metabolism of T2DM rats. PCs, LysoPCs, LysoPEs were largely involved in the pathogenesis of inflammatory and metabolic diseases such as diabetes [56]. PCs and LysoPCs could block ERK, P38 activation and NF-ƙB translocation to the nucleus in peritoneal macrophages, which had an intrinsic anti-inflammatory property [57,58,59]. Many studies also indicated that levels of lysoPCs are closely associated with oxidative stress and inflammation [60,61]. LysoPCs containing an unsaturated fatty acyl group such as C20:4 and C22:6 were reported to show potent anti-inflammatory activity in in vivo and in vitro models [62,63]. Lower levels of LysoPCs were found to be predictors for T2DM in earlier studies [64] and treatment with LysoPCs could result in a significant increase in the level of GLUT4 at the plasma membranes of 3T3-L1 adipocytes [65]. Thus, the modification of SR, CR, LSC, and HSC on LysoPCs, LysoPEs and PCs levels might contribute to the amelioration of inflammation and insulin resistance. SR and CR combined therapy exhibited better efficacy (Table S2).

By the analysis of urine metabolomics, purine metabolism was notably modified after combined therapy, however, the single drug showed no effect; purine metabolism was terribly dysregulated which was associated with T2DM [66]. Xanthosine, a nucleoside derived from the purine base xanthine and ribose, was elevated in T2DM rats and catabolism of xanthosine could ultimately lead to the production of high uric acid which was linked to diabetic complications [67,68]. Interestingly, xanthosine was affected by neither SR nor CR, but by their combination. Additionally, hippuric acid, which could be converted from dietetic aromatic compounds by gut microbes [69], was significantly decreased in urine of T2DM rats. This might be due to disturbed gut microbiota metabolism under diabetic status [70]. As reported, concentrations of hippuric acid were lower in HFD-induced hyperlipidemic rats than in normal rats [71]. d-glucurono-6,3-lactone, participating in ascorbate and aldarate metabolism, was increased in T2DM rats and was downregulated after treatment of SR, CR and their combined extracts. Pyrimidine metabolism was also disturbed and increased metabolite of deoxyuridine in the urine of T2DM rats was identified. After 30 days therapeutic intervention, these changed trends in T2DM rats were reversed by SR and CR, particularly by their combined extracts (Table S2).

## 4. Materials and Methods

### 4.1. Chemicals, Reagents, and Materials

Acetonitrile (HPLC grade) was bought from TEDIA (Fairfield, OH, USA). Formic acid was supplied by Merck KGaA (Darmstadt, Germany). Metformin was purchased from Merck Serono (Shanghai, China). SR and CR were purchased from Nanjing Guo-yao Pharm (Nanjing, Jiangsu, China). Streptozotocin (STZ) was obtained from Sigma chemical (St Louis, MO, USA).

### 4.2. Extract Preparation

The dry herb pieces of SR (1.0 kg), CR (1.0 kg) and SC (1:1, 3 kg) were extracted with boiling water (1:8) twice, 2 h each time, filtered through gauze and concentrated to 1.0 g/mL, respectively.

### 4.3. Animals and Induction of T2DM Rats

Pathogen-free male Sprague-Dawley rats (200 ± 20 g) were supplied by the Zhejiang Province Experimental Animal Center. All animals were maintained in cages at 22–26 °C with 55%–65% relative humidity, under a 12 h dark/light cycle, with water and respective diet available ad libitum. The experiments were performed following the principles of the Care and Use of Laboratory Animal and approved by the Animal Ethics Committee of Nanjing University of Chinese Medicine.

After acclimatization for one week, animals were randomly separated into seven groups (6 rats/group): normal group (N), model group (M), metformin group (P), Scutellaria Radix group (SR), Coptidis Rhizome group (CR), Low dose of combined extracts group (LSC), and High dose of combined extracts group (HSC). The normal rats were fed with common pellet diets during the experiment and rats in other groups were fed by a high-fat diet (including 67.5% standard laboratory chow, 20% sucrose, 10% lard oil, and 2.5% egg yolk powder(*w*/*w*)). After one month of dietary intervention, a dose of 30 mg/kg STZ (dissolved using 0.1 M citrate buffer, pH 4.2) was given to all the groups except the normal group. The normal rats were given the same dose of sodium buffer. Three days later, all rats were fasted 12 h (free access to water). Fasting blood glucose (FBG) was measured by ONE TOUCH II type blood sugar apparatus. Rats were considered to be T2DM model when their FBG levels exceeded 16.7 mmol/L.

### 4.4. Drug Administration, Biological Sample Collection, and Preparation

The P group rats were intragastrically given metformin at a dose of 0.09 g/kg (0.09 g metformin per 1 kg rat body weight) for one month. The SR and CR group rats were intragastrically given SR, CR extracts at a dose of 6.3 g/kg (6.3 g crude herbs per 1 kg rat body weight) for one month, respectively. The LSC and HSC group rats were intragastrically given SC extracts at a dose of 6.3 g/kg, 12.6 g/kg (6.3 g crude herbs per 1 kg rat body weight, 12.6 g crude herbs per 1 kg rat body weight) for one month, respectively. All experiments and animal care were approved by the Animal Ethics Committee of Nanjing University of Chinese Medicine.

The rats were fixed in supine position and anesthetized with 10% chloral hydrate by intraperitoneal injection, blood samples were collected in heparinized tubes and no anticoagulant tubes on the 30th day after treatment from abdominal aorta. They were then centrifuged at 3000 r/min for 10 min to obtain the plasma and serum samples stored at −80 °C. The levels of pro-inflammatory cytokines and biochemical indexes in serum were measured using Elisa kits.

Liver tissues were quickly removed, placed on ice, and homogenized in 9 vol. (*w*/*v*) of phosphate buffer saline. The homogenate was centrifuged at 4 °C, 13,000 rpm for 10 min and supernatant liquor was then stored at −80 °C until detection of PEPCK, FBPase, G6Pase, GP, GK, PFK, PK, and GS by using Elisa kits according to the instructions of the manufacturer.

One hundred microliters of plasma were added to 300 μL of acetonitrile and two hundred microliters of urine were added to 200 μL of acetonitrile. These mixtures were vortexed for 1 min and centrifuged at 13,000 r/min for 10 min to obtain the supernatant. A 2 μL aliquot of each plasma or urine sample was injected for LC/MS analysis.

### 4.5. Histological Analysis

Each liver, pancreas, skeletal muscle, adipose tissue was fixed in 4% (*w*/*v*) paraformaldehyde over 24 h. With graded ethanol dehydration, specimens were embedded in paraffin. Sequentially, 3 mm sections were rehydrated and stained with hematoxylin and eosin. The program of staining was obtained with the IX51 microscope (Olympus Corporation, Tokyo, Japan).

### 4.6. Real-Time PCR

Total RNA was isolated from individual liver of each group for analysis of P38, ERK, JNK, IKK, c-jun, c-fos, P65, IRS1, PI3K, Akt2, and Glut2 using Trizol reagent (Invitrogen, Carlsbad, CA, USA) following the protocol provided by the manufacturer. Real-time quantitative PCR was performed by using SYBR Green Master mix and Rox reference dye according to the manufacturer’s instructions. The cDNAs were obtained by the reverse transcription of RNA from rat liver. The mRNA levels of individual genes were normalized and calculated using the ∆∆CT method. The primers are listed in Table S1.

### 4.7. Western Blot

Individual liver of each group for analysis of P65, p-P65, PI3K, p-PI3K, Akt2, p-Akt2, and Glut2 was pulverized, and the proteins were extracted by a total protein extraction kit (Keygen, Shenzhen, China). An equal amount of protein (40 µg) in liver was separated by SDS-PAGE and transferred to polyvinylidene difluoride (PVDF) membranes (Millipore, Billerica, MA, USA). The membranes were blocked with 5% fat-free milk and incubated with different primary antibodies. The bound antibodies were detected using horseradish peroxidase–conjugated anti-rabbit antibodies. Antibody reactivity was detected by ECL Western blotting Detection Systems.

### 4.8. Metabolic Profiling

#### 4.8.1. Chromatography

Chromatographic experiments were performed on a Thermo Syncronis C_18_ column (2.1 mm i.d. × 100 mm, 1.7 μm) using an Acquity UPLC^TM^ System (Waters Corp., Milford, MA, USA). The column was maintained at 35 °C, and the mobile phase, at a flow rate of 0.4 mL/min, consisting of solvent A (0.1% formic acid in water) and mobile phase B (acetonitrile). 0–12 min, 5%–20% B; 12–14 min, 20%–25% B; 14–19 min, 25%–55% B; 19–21 min, 55%–70% B; 21–25 min, 70%–95% B.

#### 4.8.2. Mass Spectrometry

MS spectrometry was performed with a Waters Synapt^TM^ Q-TOF/MS (Waters Corp., Milford, CT, USA). The conditions used for the electrospray ion (ESI) source were as follows: capillary voltage of 3.0 kV, sample cone voltage of 30.0 V; extraction cone voltage of 2.0 V, source temperature of 120 °C, desolvation temperature of 350 °C. Nitrogen was used as desolvation and cone gas with a flow rate of 600 and 50 L/h, respectively. Leucine-enkephalin was used as the lock mass generating an [M + H]^+^ ion (*m*/*z* 556.2771) and [M-H]^−^ ion (*m*/*z* 554.2615) at a concentration of 200 pg/mL and flow rate of 100 μL/min to ensure accuracy during the MS analysis via a syringe pump.

#### 4.8.3. Metabolomic Data Processing and Multivariate Analysis

UPLC/MS data were detected and noise-reduced in both the UPLC and MS domains such that only true analytical peaks were selected for further processing by the MassLynx software (version 4.1, Waters Corp, Milford, MA, USA).

#### 4.8.4. Biomarker Identification and Metabolic Pathway Analysis

The identities of the potential biomarkers were confirmed by comparing their mass spectra and chromatographic retention times with the available reference standards and a full spectral library containing MS/MS data obtained in the positive and/or negative ion modes. The Mass Fragment application manager (Waters MassLynx v4.1, Waters corp., Milford, CT, USA) was used to facilitate the MS/MS fragment ion analysis through the use of chemically intelligent peak-matching algorithms. This information was then used to search multiple databases and to analyze the potential metabolic pathway using MetPA. Potential biological roles were evaluated by an enrichment analysis using MetaboAnalyst (version 3.0, McGill Univeristy, Montreal, Canada).

### 4.9. Statistical Analysis

Statistical analysis was assessed by SPSS 19.0 (SPSS Inc., Chicago, IL, USA). All data were given as mean ± SD, and comparison of mean values was evaluated using One-way-ANOVA with Dunnett. In all experiments, *p* values <0.05 were considered significant difference.

## 5. Conclusions

Previous studies suggested that macrophage-specific TGR5 signaling in kupffer cells protected liver from inflammation and insulin resistance.An increase of cholic acid and deoxycholic acid was found, while glycocholic acid was decreased in T2DM rats. So combined SR and CR could better modulate inflammation and improve insulin resistance by increasing the levels of glycocholic aid and decreasing the levels of cholic acid and deoxycholic acid. In conclusion, the present studies suggested that combined extracts exerted significant amelioration on T2DM by modulation of proinflammatory cytokines, key target protein expressions in MAPK, and insulin signaling pathways as well as enzymatic activities related to glycometabolism. Thus, administration of combined extract could improve the symptoms of T2DM rats more effectively than the single drug. These results might provide useful hints for T2DM treatment and deserve further clinical investigations.

## Supplementary Materials

Supplementary materials can be found at http://www.mdpi.com/1422-0067/19/11/3634/s1.

## Abbreviations

Akt	Protein kinase B
CR	Coptidis Rhizoma
CRP	C-reactive protein
ERK	Extracellular regulated protein kinases
FBG	Fasting blood glucose
FBPase	Fructose-1,6-bisphosphatase
FFA	Free fatty acid
FINS	Fasting insulin
G6Pase	Glucose 6-phosphatase
GK	Glucokinase
Glut 2	Glucose transporter 2
GP	Glycogen phosphorylase
GS	Glycogen synthase
HDL-C	High-density lipoprotein
HFD	High-fat diet
HSC	How dose of combined extracts group
IFN-γ	Interferon gamma
IKK	Inhibitor of nuclear factor kappa-B kinase
IL-1β	Interleukin 1β
IL-6	Interleukin 6
IRS1	Insulin receptor substrate 1
JNK	C-Jun N-terminal kinase
LDL-C	Low-density lipoprotein
LSC	Low dose of combined extracts group
MAPK	Mitogen-activated protein kinase
NO	Nitric oxide
P	Metformin
P38	P38 mitogen-activated protein kinases
PEPCK	Phosphoenolpyruvate carboxykinase
PFK	Phosphofructokinase
PI3K	Phosphatidylinositol-4,5-bisphosphate 3-kinase
PK	Pyruvate kinase
SOCS3	Suppressor of cytokine signaling 3
SR	Scutellariae Radix
STZ	Streptozocin
T2DM	Type 2 diabetes mellitus
TC	Total chelosterol
TCM	Traditional Chinese medicine
TG	Triglyceride
TNF-α	Tumor necrosis factor alpha
UPLC-Q-TOF/MS	Ultra performance liquid chromatography-quadrupole time-of-flight mass spectrometry

## References

[1] Kalteniece A., Ferdousi M., Azmi S., Marshall A., Soran H., Malik R.A. (2018). Keratocyte Density Is Reduced and Related to Corneal Nerve Damage in Diabetic Neuropathy. Investig. Ophthalmol. Vis. Sci..

[2] Cheungpasitporn W., Thongprayoon C., Vijayvargiya P., Anthanont P., Erickson S.B. (2016). The risk for new-onset diabetes mellitus after kidney transplantation in patients with autosomal dominant polycystic kidney disease: A systematic review and meta-analysis. Can. J. Diabetes.

[3] Zaoui P., Hannedouche T., Combe C. (2017). Cardiovascular protection of diabetic patient with chronic renal disease and particular case of end-stage renal disease in elderly patients. Nephrol. Ther..

[4] Aldossari K.K. (2018). Cardiovascular outcomes and safety with antidiabetic drugs. Int. J. Health Sci..

[5] Guariguata L., Whiting D.R., Hambleton I., Beagley J., Linnenkamp U., Shaw J.E. (2014). Global estimates of diabetes prevalence for 2013 and projections for 2035. Diabetes Res. Clin. Pract..

[6] Meneilly G.S., Tessier D.M. (2016). Diabetes, dementia and hypoglycemia. Can. J. Diabetes.

[7] Bonnet F., Scheen A. (2017). Understanding and overcoming metformin gastrointestinal intolerance. Diabetes Obes. Metab..

[8] Yang X., Zhang Q., Gao Z., Yu C., Zhang L. (2018). Baicalin alleviates IL-1β-induced inflammatory injury via down-regulating miR-126 in chondrocytes. Biomed. Pharmacother..

[9] Cheng C.S., Chen J., Tan HY., Wang N., Chen Z., Feng Y. (2018). Scutellaria baicalensis and cancer treatment: Recent progress and perspectives in biomedical and clinical studies. Am. J. Chin. Med..

[10] Zhang X.W., Li W.F., Li W.W., Ren K.H., Fan C.M., Chen Y.Y., Shen Y.L. (2011). Protective effects of the aqueous extract of Scutellaria baicalensis against acrolein-induced oxidative stress in cultured human umbilical vein endothelial cells. Pharm. Biol..

[11] Yang M.D., Chiang Y.M., Higashiyama R., Asahina K., Mann J., Wang C.C., Tsukamoto H. (2012). Rosmarinic acid and baicalin epigenetically depress peroxisomal proliferator-activated receptor γ in hepatic stellate cells for their antifibrotic effect. Hepatology.

[12] Song K.H., Lee S.H., Kim B.Y., Park A.Y., Kim J.Y. (2013). Extracts of Scutellaria baicalensis reduced body weight and blood triglyceride in db/db Mice. Phytother. Res..

[13] Wang T., Jiang H., Cao S., Chen Q., Cui M., Wang Z., Li D., Zhou J., Wang T., Qiu F. (2017). Baicalin and its metabolites suppresses gluconeogenesis through activation of AMPK or AKT in insulin resistant HepG-2 cells. Eur. J. Med. Chem..

[14] Sharma G., Nam J.S., Sharma A.R., Lee S.S. (2018). Antimicrobial Potential of Silver Nanoparticles Synthesized Using Medicinal Herb Coptidis rhizome. Molecules.

[15] Chai F.N., Ma W.Y., Zhang J., Xu H.S., Li Y.F., Zhou Q.D., Li X.G., Ye X.L. (2018). Coptisine from Rhizoma coptidis exerts an anti-cancer effect on hepatocellular carcinoma by up-regulating miR-122. Biomed. Pharmacother..

[16] Zhang Z., Zhang H., Li B., Meng X., Wang J., Zhang Y., Yao S., Ma Q., Jin L., Yang J. (2014). Berberine activates thermogenesis in white and brown adipose tissue. Nat. Commun..

[17] Xie H., Wang Q., Zhang X., Wang T., Hu W., Manicum T., Chen H., Sun L. (2018). Possible therapeutic potential of berberine in the treatment of STZ plus HFD-induced diabetic osteoporosis. Biomed. Pharmacother..

[18] Huang L., Yue P., Wu X., Yu T., Wang Y., Zhou J., Kong D., Chen K. (2018). Combined intervention of swimming plus metformin ameliorates the insulin resistance and impaired lipid metabolism in murine gestational diabetes mellitus. PLoS ONE.

[19] Wei X., Gu N., Feng N., Guo X., Ma X. (2018). Inhibition of p38 mitogen-activated protein kinase exerts a hypoglycemic effect by improving β cell function via inhibition of β cell apoptosis in db/db mice. J. Enzyme Inhib. Med. Chem..

[20] Pace F., Carvalho B.M., Zanotto T.M., Santos A., Guadaqnini D., Silva K.L.C., Mendes M.C.S., Rocha G.Z., Alegretti S.M., Santos G.A. (2018). Helminth infection in mice improves insulin sensitivity via modulation of gut microbiota and fatty acid metabolism. Pharmacol. Res..

[21] Jourdan T., Nicoloro S.M., Zhou Z., Shen Y., Liu J., Coffey N.J., Cinar R., Godlewski G., Gao B., Aouadi M. (2017). Decreasing CB1 receptor signaling in Kupffer cells improves insulin sensitivity in obese mice. Mol. Metab..

[22] Kim E.A., Kim S.Y., Ye B.R., Kim J., Ko S.C., Lee W.W., Kim K.N., Choi I.W., Jung W.K., Heo S.J. (2018). Anti-inflammatory effect of Apo-9′-fucoxanthinone via inhibition of MAPKs and NF-kB signaling pathway in LPS-stimulated RAW 264.7 macrophages and zebrafish model. Int. Immun. Pharmacol..

[23] Kwon E.Y., Choi M.S. (2018). Luteolin Targets the Toll-Like Receptor Signaling Pathway in Prevention of Hepatic and Adipocyte Fibrosis and Insulin Resistance in Diet-Induced Obese Mice. Nutrients.

[24] Chung S.T., Courville A.B., Onuzuruike A.U., Galvan-De La Cruz M., Mabundo L.S., DuBose C.W., Kasturi K., Cai H., Gharib A.M., Walter P.J. (2018). Gluconeogenesis and risk for fasting hyperglycemia in Black and White women. JCI Insight.

[25] Godfrey R., Quinlivan R. (2016). Skeletal muscle disorders of glycogenolysis and glycolysis. Nat. Rev. Neurol..

[26] Ros S., Zafra D., Valles-Ortega J., García-Rocha M., Forrow S., Domínguez J., Calb J., Guinovart J.J. (2010). Hepatic overexpression of a constitutively active form of liver glycogen synthase improves glucose homeostasis. J. Biol. Chem..

[27] Johnson A.M., Olefsky J.M. (2013). The origins and drivers of insulin resistance. Cell.

[28] Ajala-Lawal R.A., Aliyu N.O., Ajiboye T.O. (2018). Betulinic acid improves insulin sensitivity, hyperglycemia, inflammation and oxidative stress in metabolic syndrome rats via PI3K/Akt pathways. Arch. Physiol. Biochem..

[29] Solinas G., Becattini B. (2016). JNK at the crossroad of obesity, insulin resistance, and cell stress response. Mol. Metab..

[30] Nandipati K.C., Subramanian S., Agrawal D.K. (2017). Protein kinases: Mechanisms and downstream targets in inflammation-mediated obesity and insulin resistance. Mol. Cell. Biochem..

[31] Dou L., Wang S., Sun L., Huang X., Zhang Y., Shen T., Guo J., Man Y., Tang W., Li J. (2017). Mir-338-3p Mediates Tnf-A-Induced Hepatic Insulin Resistance by Targeting PP4r1 to Regulate PP4 Expression. Cell. Physiol. Biochem..

[32] Li X., Wang X., Wu D., Chen Z.B., Wang M.X., Gao Y.X., Gong C.X., Qin M. (2018). Interleukin-1β and C-reactive protein level in plasma and gingival crevicular fluid in adolescents with diabetes mellitus. Beijing Da Xue Xue Bao Yi Xue Ban.

[33] Zhou M.M., Zhang W.Y., Li R.J., Guo C., Wei S.S., Tian X.M., Luo J., Kong L.Y. (2018). Anti-inflammatory activity of Khayandirobilide A from Khaya senegalensis via NF-κB, AP-1 and p38 MAPK/Nrf2/HO-1 signaling pathways in lipopolysaccharide-stimulated RAW 264.7 and BV-2 cells. Phytomedicine.

[34] Van Poelje P.D., Potter S.C., Erion M.D. (2011). Fructose-1, 6-bisphosphatase inhibitors for reducing excessive endogenous glucose production in type 2 diabetes. Handb. Exp. Pharmacol..

[35] Liu Q., Zhang F.G., Zhang W.S., Pan A., Yang Y.L., Liu J.F., Li P., Liu B.L., Qi L.W. (2017). Ginsenoside Rg1 inhibits glucagon-induced hepatic gluconeogenesis through Akt-FoxO1 interaction. Theranostics.

[36] Pari L., Chandramohan R. (2017). Modulatory effects of naringin on hepatic key enzymes of carbohydrate metabolism in high-fat diet/low-dose streptozotocin-induced diabetes in rats. Gen. Physiol. Biophys..

[37] Hermida M.A., Dinesh Kumar J., Leslie N.R. (2017). GSK3 and its interactions with the PI3K/AKT/mTOR signalling network. Adv. Biol. Regul..

[38] Zhang X., Jin Y., Wu Y., Zhang C., Jin D., Zheng Q., Li Y. (2018). Anti-hyperglycemic and anti-hyperlipidemia effects of the alkaloid-rich extract from barks of *Litsea glutinosa* in ob/ob mice. Sci. Rep..

[39] Bockus L.B., Matsuzaki S., Vadvalkar S.S., Young Z.T., Giorgione J.R., Newhardt M.F., Kinter M., Humphries K.M. (2017). Cardiac Insulin Signaling Regulates Glycolysis Through Phosphofructokinase 2 Content and Activity. J. Am. Heart. Assoc..

[40] Wang S., Yu X., Zhang W., Ji F., Wang M., Yang R., Li H., Chen W., Dong J. (2018). Association of serum metabolites with impaired fasting glucose/diabetes and traditional risk factors for metabolic disease in Chinese adults. Clin. Chim. Acta.

[41] Choi E., Zhang X., Xing C., Yu H. (2016). Mitotic checkpoint regulators control insulin signaling and metabolic homeostasis. Cell.

[42] Huypens P., Sass S., Wu M., Dyckhoff D., Tschöp M., Theis F., Marschall S., Hrabě de Angelis M., Beckers J. (2016). Epigenetic germline inheritance of diet-induced obesity and insulin resistance. Nat. Genet..

[43] Jang C., Oh S.F., Wada S., Rowe G.C., Liu L., Chan M.C., Rhee J., Hoshino A., Kim B., Ibrahim A. (2016). A branched-chain amino acid metabolite drives vascular fatty acid transport and causes insulin resistance. Nat. Med..

[44] Wang S., Deng Y., Xie X., Ma J., Xu M., Zhao X., Gu W., Hong J., Wang W., Xu G. (2018). Plasma bile acid changes in type 2 diabetes correlated with insulin secretion in two-step hyperglycemic clamp. J. Diabetes.

[45] Nowak C., Hetty S., Salihovic S., Castillejo-Lopez C., Ganna A., Cook N.L., Broeckling C.D., Prenni J.E., Shen X., Giedraitis V. (2018). Glucose challenge metabolomics implicates medium-chain acylcarnitines in insulin resistance. Sci. Rep..

[46] Zaborska K.E., Cummings B.P. (2018). Rethinking Bile Acid Metabolism and Signaling for Type 2 Diabetes Treatment. Curr. Diabetes Rep..

[47] Qiao X., Ye M., Xiang C. (2012). Metabolic regulatory effects of licorice: A bile acid metabonomic study by liquid chromatography coupled with tandem mass spectrometry. Steroids.

[48] Agarwal S., Sasane S., Kumar J., Deshmukh P., Bhayani H., Giri P., Giri S., Soman S., Kulkarni N., Jain M. (2018). Evaluation of novel TGR5 agonist in combination with Sitagliptin for possible treatment of type 2 diabetes. Bioorg. Med. Chem. Lett..

[49] Kumar D.P., Rajagopal S., Mahavadi S., Mirshahi F., Grider J.R., Murthy K.S., Sanyal A.J. (2012). Activation of transmembrane bile acid receptor TGR5 stimulates insulin secretion in pancreatic β cells. Biochem. Biophys. Res. Commun..

[50] Zhang X., Wall M., Sui Z., Kauffman J., Hou C., Chen C., Du F., Kirchner T., Liang Y., Johnson D.L. (2017). Discovery of Orally Efficacious Tetrahydrobenzimidazoles as TGR5 Agonists for Type 2 Diabetes. ACS Med. Chem. Lett..

[51] Lo S.H., Li Y., Cheng K.C., Niu C.S., Cheng J.T., Niu H.S. (2017). Ursolic acid activates the TGR5 receptor to enhance GLP-1 secretion in type 1-like diabetic rats. Naunyn Schmiedebergs Arch. Pharmacol..

[52] Yoneno K., Hisamatsu T., Shimamura K., Kamada N., Ichikawa R., Kitazume M.T., Mori M., Uo M., Namikawa Y., Matsuoka K. (2013). TGR5 signaling inhibits the production of pro-inflammatory cytokines by in vitro differentiated inflammatory and intestinal macrophages in Crohn’s disease. Immunology.

[53] Pols T.W., Nomura M., Harach T., Lo Sasso G., Oosterveer M.H., Thomas C., Rizzo G., Gioiello A., Adorini L., Pellicciari R. (2011). TGR5 activation inhibits atherosclerosis by reducing macrophage inflammation and lipid loading. Cell Metab..

[54] Zeng H., Botnen J.H., Briske-Anderson M. (2010). Deoxycholic acid and selenium metabolite methylselenol exert common and distinct effects on cell cycle, apoptosis, and MAP kinase pathway in HCT116 human colon cancer cells. Nutr. Cancer.

[55] Kuno T., Hirayama-Kurogi M., Ito S., Ohtsuki S. (2018). Reduction in hepatic secondary bile acids caused by short-term antibiotic-induced dysbiosis decreases mouse serum glucose and triglyceride levels. Sci. Rep..

[56] Zeng H., Tong R., Tong W., Yang Q., Qiu M., Xiong A., Sun S., Ding L., Zhang H., Yang L. (2017). Metabolic Biomarkers for Prognostic Prediction of Pre-diabetes: Results from a longitudinal cohort study. Sci. Rep..

[57] Carneiro A.B., Iaciura B.M., Nohara L.L., Lopes C.D., Veas E.M., Mariano V.S., Bozza P.T., Lopes U.G., Atella G.C., Almeida I.C. (2013). Lysophosphatidylcholine triggers TLR2- and TLR4-mediated signaling pathways but counteracts LPS-induced NO synthesis in peritoneal macrophages by inhibiting NF-ƙB translocation and MAPK/ERK phosphorylation. PLoS ONE.

[58] Treede I., Braun A., Jeliaskova P., Giese T., Füllekrug J., Griffiths G., Stremmel W., Ehehalt R. (2009). TNF-alpha-induced up-regulation of pro-inflammatory cytokines is reduced by phosphatidylcholine in intestinal epithelial cells. BMC Gastroenterol..

[59] Treede I., Braun A., Sparla R., Kühnel M., Giese T., Turner J.R., Anes E., Kulaksiz H., Füllekrug J., Stremmel W. (2007). Anti-inflammatory effects of phosphatidylcholine. J. Biol. Chem..

[60] Suvitaival T., Bondia-Pons I., Yetukuri L., Pöhö P., Nolan J.J., Hyötyläinen T., Kuusisto J., Orešič M. (2018). Lipidome as a predictive tool in progression to type 2 diabetes in Finnish men. Metabolism.

[61] Schilling T., Eder C. (2010). Importance of lipid rafts for lysophosphatidyl choline-induced caspase-1 activation and reactive oxygen species generation. Cell. Immunol..

[62] Huang L.S., Hung N.D., Sok D.E., Kim M.R. (2010). Lysophosphatidylcholine containing docosahexaenoic acid at the sn-1 position in anti-inflammatory. Lipids.

[63] Hung N.D., Kim M.R., Sok D.E. (2011). Mechanisms for anti-inflammatory effects of 1-[15(S)-hydroxyeicosapentaenoyl] lysophosphatidylcholine, administered intraperitoneally, in zymosan A-induced peritonitis. Br. J. Pharmacol..

[64] Wang-Sattler R., Yu Z., Herder C. (2012). Novel biomarkers for pre-diabetes identified by metabolomics. Mol. Syst. Biol..

[65] Yea K., Kim J., Yoon J.H., Kwon T., Kim J.H., Lee B.D., Lee H.J., Lee S.J., Kim J.I., Lee T.G. (2009). Lysophosphatidylcholine activates adipocyte glucose uptake and lowers blood glucose levels in murine models of diabetes. J. Biol. Chem..

[66] Dudzinska W. (2014). Purine nucleotides and their metabolites in patients with type 1 and 2 diabetes mellitus. J. Biomed. Sci. Eng..

[67] Kushiyama A., Tanaka K., Hara S., Kawazu S. (2014). Linking uric acid metabolism to diabetic complications. World J. Diabetes.

[68] Zoppini G., Targher G., Chonchol M., Ortalda V., Abaterusso C., Pichiri I., Negri C., Bonora E. (2012). Serum uric acid levels and incident chronic kidney disease in patients with type 2 diabetes and preserved kidney function. Diabetes Care.

[69] Xiao Y., Dong J., Yin Z., Wu Q., Zhou Y., Zhou X. (2018). Procyanidin B2 protects against d-galactose-induced mimetic aging in mice: Metabolites and microbiome analysis. Food. Chem. Toxicol..

[70] Li L., Wang C., Yang H., Liu S., Lu Y., Fu P., Liu J. (2017). Metabolomics reveal mitochondrial and fatty acid metabolism disorders that contribute to the development of DKD in T2DM patients. Mol. Biosyst..

[71] Wu Q., Zhang H., Dong X., Chen X.F., Zhu Z.Y., Hong Z.Y., Chai Y.F. (2014). UPLC-Q-TOF/MS based metabolomic profiling of serum and urine of hyperlipidemic rats induced by high fat diet. J. Pharm. Anal..

