# Supplementary material for: Scutellariae Radix and Coptidis Rhizoma Improve Glucose and Lipid Metabolism in T2DM Rats via Regulation of the Metabolic Profiling and MAPK/PI3K/Akt Signaling Pathway
Source: Int J Mol Sci. 2018 Nov 18;19(11):3634. doi: 10.3390/ijms19113634 (PMC6274950; doi:10.3390/ijms19113634)
Supplement: Supplementary file 1 [file ijms-19-03634-s001.pdf]

Table S1. Primer sequences used in real-time PCR.

mRNA	Primer	Sequence	Accession Number
JNK	Forward	CTGAAGCAGAAGCTCCACCA	XM_012812663.2
	Reverse	GCTGCCCCCGTATAACTCC	
P65	Forward	CCATACGCTGACCCTAGCCT	NM_199267.2
	Reverse	CGATCGTCTGTGTCTGGCAAGT	
IRS1	Forward	CGCTACATCCCAGGTGCTAC	NM_012969.1
	Reverse	GAAACCACTGAGGACTGCGA	
IKK	Forward	GGAGTTTGGCATCACATCGG	AF_115282.1
	Reverse	ATCGGGCTCCTCTGTAGGTC	
PI3K	Forward	ATGACAAGGAACAGCTCCGA	NM_133399.2
	Reverse	GCAGTACATCTGGGCCACTT	
Akt2	Forward	CAGGCACCCCTTCCTTACAG	NM_017093.1
	Reverse	GGTACACCACATCCGTCGAG	
Glut2	Forward	TCAGATGAAGGCCACCCATT	NM_031831.1
	Reverse	AACACCCACATCAACACTGC	
P38	Forward	CTGCGAGGGCTGAAGTAT	XM_003803280.3
	Reverse	TCCTCTTATCCGAGTCCAA	
ERK	Forward	GGACCTGAAGCCCTCCAATC	NM_017347.2
	Reverse	CCAGAACCGTCTACCAGAGC	
C-fos	Forward	GGCAGAAGGGGCAAAGTAGA	NM_022197.2
	Reverse	AGTTGATCTGTCTCCGCTTGG	
C-jun	Forward	CGCCCTAGCTGAACTGCATA	NM_021835.3
	Reverse	AAGTTGCTGAGGTTGGCGTA	

Table S2. Potential biomarkers of P, SR, CR, LSC and HSC treated diabetic rats.

No	t_R/min	$[M\pm H]^{\pm}$	Metabolites	Model	Effects of test drugs					Pathway	Resource	Ion mode
				M/N	P	SR	CR	LSC	HSC			
1	4.39	407.2792	Cholic acid	$\uparrow^{\#\#\#}$	$\downarrow^{***}$	$\downarrow^{*}$	$\downarrow^{*}$	$\downarrow^{**}$	$\downarrow^{***}$	Primary bile acid biosynthesis	plasma	ESI-
2	6.00	391.2849	Deoxycholic acid	$\uparrow^{\#\#\#}$	$\downarrow^{***}$	$\downarrow^{*}$	$\downarrow^{*}$	$\downarrow^{**}$	$\downarrow^{***}$	Primary bile acid biosynthesis	plasma	ESI-
3	7.26	480.3092	LysoPE(0:0/18:0)	$\downarrow^{\#\#\#}$	$\uparrow^{***}$	$\uparrow^{*}$	$\uparrow^{*}$	$\uparrow^{**}$	$\uparrow^{**}$	Glycerophospholipid metabolism	plasma	ESI-
4	7.61	540.3302	LyspPC(16:0)	$\downarrow^{\#\#\#}$	$\uparrow^{***}$	$\uparrow^{*}$	$\uparrow^{*}$	$\uparrow^{*}$	$\uparrow^{**}$	Glycerophospholipid metabolism	plasma	ESI-
5	6.70	504.3101	LysoPE(20:2(11Z,14Z)/0:0)	$\downarrow^{\#\#\#}$	$\uparrow^{***}$	$\uparrow^{*}$	$\uparrow^{*}$	$\uparrow^{*}$	$\uparrow^{**}$	Glycerophospholipid metabolism	plasma	ESI-
6	4.30	283.0688	Xanthosine	$\uparrow^{\#\#}$	$\downarrow^{**}$	$\downarrow$	$\downarrow$	$\downarrow^{*}$	$\downarrow^{**}$	Purine metabolism	urine	ESI-
7	5.94	201.0219	Hippuric acid	$\downarrow^{\#\#}$	$\uparrow^{**}$	$\uparrow^{*}$	$\uparrow$	$\uparrow^{*}$	$\uparrow^{**}$	Phenylalanine metabolism	urine	ESI-
8	3.22	222.0806	N-acetyl-L-tyrosine	$\downarrow^{\#\#}$	$\uparrow^{**}$	$\uparrow$	$\uparrow^{*}$	$\uparrow^{*}$	$\uparrow^{**}$	Tyrosine metabolism	urine	ESI-
9	3.60	175.0243	D-glucurono-6,3-lactone	$\uparrow^{\#\#}$	$\downarrow^{**}$	$\downarrow^{*}$	$\downarrow^{*}$	$\downarrow^{*}$	$\downarrow^{**}$	Ascorbate and aldarate metabolism	urine	ESI-
10	6.92	520.3410	LysoPC(18:2(9z,12z))	$\downarrow^{***}$	$\uparrow^{**}$	$\uparrow^{*}$	$\uparrow^{*}$	$\uparrow^{*}$	$\uparrow^{**}$	Glycerophospholipid metabolism	plasma	ESI+
11	12.82	758.5731	PC(16:1(9z)/18:1(11z))	$\downarrow^{***}$	$\uparrow^{**}$	$\uparrow^{*}$	$\uparrow^{*}$	$\uparrow^{*}$	$\uparrow^{**}$	arachidonic acid metabolism	plasma	ESI+
12	6.41	494.3239	LysoPC(16:1(9z))	$\downarrow^{***}$	$\uparrow^{**}$	$\uparrow^{*}$	$\uparrow^{*}$	$\uparrow^{*}$	$\uparrow^{**}$	Glycerophospholipid metabolism	plasma	ESI+
13	6.00	468.3096	Glycocholic acid	$\downarrow^{***}$	$\uparrow^{**}$	$\uparrow^{*}$	$\uparrow^{*}$	$\uparrow^{**}$	$\uparrow^{**}$	Primary bile acid biosynthesis	plasma	ESI+
14	1.01	229.1525	Deoxyuridine	$\uparrow^{***}$	$\downarrow^{**}$	$\downarrow^{*}$	$\downarrow^{*}$	$\downarrow^{*}$	$\downarrow^{**}$	Pyrimidine metabolism	urine	ESI+

↑ indicates increase; ↓ indicates decrease. N: Normal group; M: Model group; P: metformin group; SR: Scutellaria Radix group; CR: Coptidis Rhizome group; LSC: Low dose of combined extracts group; HSC: High dose of combined extracts group. # indicates significant change of M *VS* N (###, $P<0.001$; ##, $P<0.01$; #， $P<0.05$); * Indicates significant change of different treatment groups *VS* M (*** $p<0.001$; ** $p<0.01$; * $p<0.05$).
